# Household Hints and Recipes

**Published:** 1884-10

**Authors:** 


CIDER VINEGAR.—A French method for converting cider into vinegar is as follows: Scald three barrels or casks with hot water, rinse thoroughly and empty. Then scald with boiling vinegar, rolling the barrels and allowing them to stand on their sides two or three days, until they become thoroughly saturated with the vinegar. The barrels are then filled about one-third with strong, pure cider vinegar and two gallons of cider added. Every eighth day thereafter two gallons of cider are added until the barrels are two-thirds full. The whole is allowed to stand fourteen days longer, when it may be found to be good vinegar, and one-half of it may be drawn and the process of filling up with cider be begun again. In summer the barrels are allowed to stand exposed to the sun, and in cold weather kept where the temperature is eighty degrees.

### Household Hints & Recipes.

POISONOUS FLY PAPER may be made by soaking coarse brown paper in a saturated water solution of white arsenic ; glucose or sugar may be added to make the paper more attractive to the flies.—*Druggists' Circular.*

DESTROYING ANTS.—Powdered borax, which drives away or destroys cockroaches, is said also to be equally obnoxious to ants. Crude petroleum, kerosene oil and naphtha are also recommended for keeping them away. The surest plan is, if possible, to deluge their headquarters with boiling water.—*Druggist's Circular.*

BLEACHING SPONGES WITH BROMINE WATER.—Water saturated with bromine (about 3 per cent.) is said to bleach sponges as effectively and to do less injury than chlorine, by simple immersion of the sponges for several hours. two treatments are usually necessary. Then rinse in dilute sulphuric acid, followed by repeated washing in cold water.—*From Annales Industrielles.*

TO REMOVE RUST SPOTS FROM NICKEL-PLATED SKATES.—Cover the spots for several days with lard. Then rub it off well, using ammonia to remove it thoroughly. If the rust is deep, then use oxalic acid solution or weak hydrochloric acid, allowing it to stay on the spots only a moment, then sponging off with water and rubbing with rotten stone or rouge. Repeat as often as necessary.—*Druggists' Circular.*

PAPER BOTTLES are now made on a large scale in Germany and Austria. The paper must be well sized. The following is said to be a good recipe for the paper: Ten parts of rags, forty of straw, fifty of brown wood pulp. The paper is impregnated or coated on both sides with sixty parts of defibrinated fresh blood, thirty-five parts of lime powder, five parts sulphate of alumina. After drying, ten or twelve rolled leaves are coated again, placed over each other, and then placed in heated monlds. The albumen in the blood forms a combination on pressure with the lime which is perfectly proof against spirits, etc. The bottles are made in two pieces, which are joined afterwards.—*American Druggist.*

A GERMAN TEST FOR WATERED MILK consists in dipping a well-polished knitting-needle into a deep vessel of milk, and then immediately withdrawing it in an upright position. If the milk is pure, a drop of the fluid will hang to the needle ; but the addition of even a small proportion of water will prevent the adheasion of the drop.—*American Druggist.*

FLAXSEED LEMONADE.—What is known as flaxseed lemonade is made as follows :

R.

Whole flaxseed, 1 ounce.
Water, boiling, 1 quart.
Juice of two lemons.
Sugar to sweeten.

Pour the boiling water on the flaxseed, in a suitable vessel, let it steep three hours, pour off the clear liquid, add the lemon-juice, and sweeten to taste. Ice it for drinking. Good for colds.—*Druggists' Circular.*

READY WAY TO DETECT TRICHINÆ IN MEAT.—Slices, two or three millimeters (one-twelfth to one-eighth in.) in thickness, are taken from several different parts of the meat to be examined. The pieces are preferably taken from the surface of the muscular portion of the meat. A series of thin sections are made of each of the pieces, and these are all plunged into a solution composed of methyl green 1 gramme (15 grains), distilled water 20 grammes (1 ounce). After about ten minutes maceration the sections are taken out, and placed to decolorize in a large vessel filled with distilled water. They remain there about half an hour, the water being agitated and changed two or three times. Finally, the water having become quite limpid, it is stirred up with a glass rod, interposing the vessel between the eye and the light, when the sections containing the trichinæ are distinguished quite readily with the naked eye. The trichinæ appear in the form of small, elongated particles, of a fine blue color. The methyl green becomes fixed to the cysts of the trichinæ with greater tenacity than to the other parts of the tissue It suffices then to examine the sections with a magnification of fifty diameters to distinguish the worm which will be found in the cyst. If, in following this method, no trichinæ are found, it is positive assurance that the meat is not infected with them.—*Am. Microscopical Journal.*

INDIA RUBBER CEMENT.—The ordinary rubber cement which is so much used by fine shoemakers is made by dissolving a quantity of gutta-percha in chloroform or carbon bisulphide until the solution has the consistency of honey. Thin down the parts to be cemented, then spread a small quantity of the cement well over the parts to be joined. Warm the parts over a flame or fire for half a minute, bring the surface to be united together, and hammer well or clamp firmly. The cement dries in a few minutes.—*American Druggist.*

---

SIMPLE PROCESS FOR PURIFYING OILS.—According to the *Corps Gras Industriels*, oils which hold in solution fatty acids or other substances may be easily purified by the process of Viallis Freres, in being filtered through sawdust impregnated with a solution of soda. If a certain quantity of oil has to be purified barrels sawn in two can be used, the bottoms of which are pierced with holes. In the bottom is placed a layer of flannel on which a layer of sawdust is placed, six or eight inches in thickness. If a colorless oil is desired, a thin layer of animal black is placed upon the sawdust. By placing two or three of these vats above each other a perfectly pure oil is obtained.

---

FIRE PROOF ENTRANCES.—There is nothing yet invented that can be said to be absolutely fireproof which affords protection to window spaces or doorways, but the heavy wooden door or shutter encased in tin has undoubted advantages. If two thicknesses of inch board —pine being preferable, because it does not warp—are nailed crosswise and fully encased in tin, locked and soldered, and thoroughly nailed under the locking, the outer surface of the wood under the tin will be speedily reduced to charcoal by the action of the heat, through the combustion of the small amount of oxygen under the tin. The charcoal then becomes a very effective non-conductor of heat, and, if the tin is tight, so that no further supply of oxygen reaches the unburnt wood beneath the charcoal, it will remain cool and strong for some hours, thus giving time to control the fire were it starts. Iron doors and shutters, on the contrary, when exposed to severe heat, become so quickly warped and twisted as to be practically useless as safeguards.—*American Druggist.*

SHELLAC SPIRIT FOR HANGING PAPER.—The Germans are utilizing a discovery for hanging paper on damp walls. It consists of coating a lining-paper on one side with a solution of shellac spirit, of somewhat greater consistency than the ordinary "French polish," and then hanging it with the side thus treated to the damp wall. The paper-hanging is then performed in the usual manner with paste. Any other resin that is equally soluble in spirits may be used in place of the shellac. According to the representations this process is found equally effective in preventing the penetration of dampness.—*American Stationer.*

---

RUBBER CEMENT.—Rubber may be cemented to iron by the following method: Mix one part of powdered shellac with ten parts of strongest water of ammonia, and shake the mixture occasionally during three or four weeks, keeping it well stoppered, until it becomes liquid, when it will be ready for use. Apply a coat of the solution to the surface of the rubber which is to be cemented. This will cause the rubber to soften, but it will become hard again when the ammonia evaporates. While the rubber is soft, apply it to the substance—glass or metal—and exert a steady pressure. It will then become firmly attached to it. Another good cement for this purpose is marine glue, which we have repeatedly described.—*American Druggist.*

---

A NEW LIQUID GLUE.—A solution of 1 part of sugar in 3 parts of water, when applied to paper, gives to the latter neither lustre nor binding properties; but if to the solution be added ¼ part of slaked lime (calcium hydrate) and the mixture, after heating to 145·165° F., be macerated for a few days, with frequent agitation, most of the lime will dissolve, and the syrupy solution possesses both adhesive and glazing properties. If to 12 to 15 parts of this solution be further added 3 parts of lime, in small fragments, the latter will rapidly dissolve on warming and, on cooling, will remain liquid without losing its adhesiveness. According to the quantity of saccharate of lime present, the consistence of the liquid glue will vary; but in all cases it will have a strong adhesive power. This liquid glue may be used for a variety of purposes, but must not be employed in presence of coloring matters, which are decomposed by lime, such as Prussian blue, zinc-green, etc.—*Polytech. Notizblatt.*

BLONDE HAIR.—Bleaching with peroxide of hydrogen so modifies or destroys the pigment upon which the natural color of the hair depeds that restoration is impossible. Ladies desiring to return to the natural color which their hair had before bleaching have simply to cease using the bleaching liquid and wait until their hair grows. If the difference in color is strongly marked, it might be advisable to cut off the blonde hair, or color it with some silver or bismuth hair dye. Ladies should not bleach their hair unless they desire to continue blondes. Qneries cannot be answered by mail.—*Druggist's Circular.*

PASTEURIZING BEER.—According to Behrend, yeast cells are not killed by warming; the author, on the contrary, finds that at 55° all the cells are killed. Yeast killed at 55° placed in a food solution with addition of fresh malt-extract, did not show a single living cell after forty hours at a temperature of 6-7°. Every single bottle of beer should be heated uniformly for a considerable time to the necessary temperature, say 2 3° over 55°. Badly pasteurized beer remains clear when kept cool, but the warmth of a room turns it turbid from formation of yeast, bacteria, fungi, albuminate and some hop resin. The turbidity of well pasteurized beer the author finds to be due to a separation of gluten; by heating such beer to 50° it will again become clear, and remain so for a long time.—M. SCHWARZ in *Bied. Centr.*

THE COSMETICS OF THE MARKET.—Part of a paper by Dr. James P. Tuttle, of New York, in *Medical Record:*

POWDERS,

| Preparation. | Main Constituents. |
|---|---|
| Pearl White | Subnitrate bismuth. |
| Flake White | Carbonate lead. |
| Saunders' Face Powder | Oxide of Zinc. |
| Complexion Powder | Bismuth subcarbon. |
| Riker's Face Powder | Carbon. calcium and zinc. |

LOTIONS.

| | |
|---|---|
| Circassian Cream | Corrosive sublimate. |
| Kalydor | Corrosive sublimate, and potash. |
| Milk of Roses | Corrosive sublimate, rose water, and oil of almond. |

ENAMELS.

| | |
|---|---|
| Laird's Bloom of Youth | Oxide zinc and calcium. |
| French's Grease Paint | Oxide ztnc and calcium. |
| Gouraud's Oriental Cream | *Calomel and water.* |
| Hagan's Magnolia Balm | Oxide zinc. |
| Bradford's Enameline | Oxide zinc. |
| Eugenie's Favorite | Carbonate lead. |
| Snow White Enamel | Carbonate lead. |
| Snow White Oriental Cream | Carbonate lead. |

A TEST FOR FRUIT SYRUPS.—The *Magazine of Pharmacy* gives the following test: Genuine fruit syrups lose their color by chlorine. Those colored with aniline derivatives give at the same time a flocculent precipitate similar to that produced by ammonia in solutions of ses quioxide of iron. Sulphurous acid destroys the color of both; sulphuric, hydrochloric, and nitric acids render the color of genuine syrups brighter and change the artificial ones into yellowish orange. Potassa decolorizes fuch sine syrups while red fruit syrups acquire a dirty greenish hue. Carbonate of potash does not change the color of artificial syrups, while the others are colored green. Basic acetate of lead gives with real fruit syrups a greenish precipitate, with fuchsine syrups a red one.

HONEY OF ROSE.—M. Silvio Plevani, pharmacist of the Hospital of Ponte Vico, at Brsscia, suggests the following as an improvement on the formula of Mel Rosæ:

Petals of fresh roses, 125 parts.
White sugar, 50 parts.

Beat together by gradual additions in a marble mortar with a wooden pestle. Add

White honey, 350 parts.
Water, 100 parts.
Distilled rose water, 50 parts.

Mix, heat in a sand bath, and afterwards express. Let it stand and deposit, and decant.

This yields a preparation of a fine rose color, with an astringent and acid taste, and marked odor. Kept in a well-stoppered bottle it may be preserved in good condition for a year.

NEURALGIA PENCILS.—So-called neuralgia pencils are now being offered by a number of German pharmacists, especially in Berlin. They are said to consist essentially of a menthol, thymol, and eucalyptol, fused and cast in small conical pellets, which are fitted in a suitable handle. When the forehead and temples are touched with the pencil, a slight impression of burning is at first produced, and soon gives way to a pleasant, cool sensation. Several pharmacists claim priority in the invention. Friedlander exhibited neuralgia pencils at the late Vienna Exhibition, and a year ago "nerve crystals" were offered by Blaser, which were described in the *Pharmaceutische Zeitung* as consisting of a mixture of crystallized Japanese peppermint oil and camphor.—*Med. Record.*

GLUE, PASTE, OR MUCILAGE.—A liquid paste or glue from starch is made by placing five pounds of potato starch in six pounds (three quarts) of water, and add one-quarter pound of pure nitric acid. Keep it in a warm place, stirring frequently for forty-eight hours. Then boil the mixture until it forms a thick and translucent substance. Dilute with water if necessary, and filter through a thick cloth.

At the same time, another is made from sugar and gum arabic. Dissolve five pounds gum arabic and one pound of sugar in five pounds of water, and add one ounce of nitric acid and heat to boiling ; then mix the above with the starch paste. The resultant paste is liquid, does not mould, and dries on paper with a gloss.

It is useful for labels, wrappers, and fine bookbinders' use.

Dry pocket glue is made from twelve parts of glue and five parts of sugar. The glue is boiled until entirely dissolved, the sugar dissolved in the hot glue, and the mass evaporated until it hardens on cooling. The hard substance dissolves rapidly in lukewarm water, and is an excellent glue for use on paper.—*American Druggist.*

---

NEW PROCESS FOR PRESERVING MEAT.—Mr. Richard Jones, who has for many years devoted his attention to the preservation of meat, has now adopted a new process. The principle consists in the injection of a fluid preparation of boracic acid into the blood of the animal immediately after it has been stunned, and before its heart has ceased to beat ; the whole operation, including the removal of the blood and chemical fluid from the body of the animal, only taking a few minutes. The quantity of boracic acid used is very small, and that little is almost immediately drawn out again with the blood. The preservation of the flesh is said to be thoroughly effected ; the quantity of the chemical left in the flesh must therefore be very small, and can scarcely be injurious to the human system ; for, as Professor Barff has proved by experiment, living animals, either of the human or other species, do not seem to be injured in any way by the consumption of it. A demonstration of the effects of the process was given in April at the Adelphi Hotel, London, when the joints cut from a sheep that had been hanging for more than seven weeks at the house of the Society of Arts were cooked in various ways, and those present agreed that the meat was equal to ordinary butcher's meat. —*Scient. Amer.*

WEIGHT AND HEIGHT OF MAN.—It is well that all persons should know what the normal weight of man really is. The following shows the relative height and weight of individuals measuring five feet and upwards :

Five feet and one inch should be 120 pounds.
Five feet two inches, should be 126 pounds.
Five feet three inches, should be 133 pounds.
Five feet four inches, should be 136 pounds.
Five feet five inches, should be 142 pounds.
Five feet six inches, should be 145 pounds.
Five feet seven inches, should be 148 pounds.
Five feet eight inches, should be 155 pounds.
Five feet nine inches, should be 162 pounds.
Five feet ten inches, should be 169 pounds.
Five feet eleven inches, should be 174 pounds.
Six feet should be 178 pounds.—*American Druggist.*

---

PREPARING PAPER FOR COPYING.—A new method or process for treating paper so as to render it permanently moist for copying purposes has been devised. In preparing the paper, one pound of salt known as chloride of magnesium is dissolved in a moderate quantity of cold or warm water, and it is ready for use. From one-half a pound to a pound of water to the pound of chloride of magnesium is said to be the most desirable amount, but more or less can be used according to circumstances. Apply this solution to sheets of ordinary copying paper, whether in book form or otherwise, by applying the compound to cloth pads well saturated with the liquids, and then place the pads between any suitable number of leaves ; then apply a pressure, at first very moderate, until the absorption of the paper is complete ; then remove the cloth pads, and apply under the press a strong pressure, and the books or sheets of paper so treated are ready for copying purposes, the use of the solution of chloride of mrgnesium being the radical or base of this invention. In all cases use copying inks, or fluids, which are preferable. Paper prepared by this method will remain permanently moist at any ordinary temperature. This method is said to be a great improvement as well as more economical than methods heretofore used. There is a great saving in the cost of materials, time and labor, it only costing about one-eighth as much to treat paper by this process. Sheets of paper prepared by this method will not adhere to each other, as is frequently the case where the paper is prepared by methods where compounds composed mainly of glycerine are used; and hence paper prepared by the method or process described is more suitable and convenient for copying purposes and will take a very clear and distinct copy. The process is patented.—*Printers' Circular.*

